# Population Dynamics and Genetic Diversity of C4 Strains of Human Enterovirus 71 in Mainland China, 1998–2010

**DOI:** 10.1371/journal.pone.0044386

**Published:** 2012-09-12

**Authors:** Dawei Guan, Sabine van der Sanden, Hanri Zeng, Wei Li, Huanying Zheng, Cong Ma, Juan Su, Zheng Liu, Xue Guo, Xin Zhang, Leng Liu, Marion Koopmans, Changwen Ke

**Affiliations:** 1 Key Laboratory of Pathogen Detection for Emergency Response of Guangdong Province, Center for Disease Control and Prevention of Guangdong Province, Guangzhou, Guangdong, China; 2 National Institute for Public Health and the Environment (RIVM), Bilthoven, The Netherlands; Centers for Disease Control and Prevention, United States of America

## Abstract

**Background:**

Since 1997, several countries within the Asian Pacific region have been affected by one or more massive outbreaks of Hand Foot and Mouth Disease (HFMD). Virus typing experiments revealed that these outbreaks were caused by strains of human enterovirus 71 (EV71) belonging to several different, recently emerged subgenogroups. In mainland China, a different situation was observed. The first outbreak, localized in Shangdong Province, was reported in 2007, and was followed by a wide-spread outbreak in mainland China in 2008. Since then, numbers of reported HFMD cases have been persistently high.

**Methodology/Principal Findings:**

To gain insight in the epidemiological behavior of EV71 in China, we studied genetic diversity and EV71 population dynamics to address whether the increase in number of reported EV71 infections reflects a real increase in viral spread or is just the result of increased awareness and surveillance. We used systematically collected VP1 gene sequences of 257 EV71 strains collected in Guangdong province from 2008 to 2010 as part of HFMD surveillance activities, and supplemented them with 305 GenBank EV71 reference stains collected in China from 1998 to 2010. All isolates from Guangdong Province belonged to subgenogroup C4. Viral population dynamics indicated that the increased reporting of HFMD in China since 2007 reflects a real increase in viral spread and continued replacement of viral lineages through time. Amino acid sequence comparisons revealed substitution of amino acid in residues 22, 145 and 289 through time regularly with the VP1 gene of EV71 strains isolated in mainland China from 1998 to 2010.

**Conclusions:**

EV71 strains isolated in mainland China mainly belonged to subgenogroup C4. There was exponential growth of the EV71 virus population in 2007 and 2008. There was amino acid substitution through time regularly with the VP1 gene which possibly increased viral spread and/or ability of the virus to circulate persistently among the Chinese population.

## Introduction

Human enterovirus 71, belonging to the *Human enterovirus A* species of the genus *Enterovirus* of the family *Picornaviridae*, is a major causative agent of hand, foot and mouth disease (HFMD) (usually in children aged <5 years) [1], [2]. Different from other enteroviruses causing HFMD, like Coxsackievirus A16, infections with EV71 can progress to severe neurological disease, including brainstem encephalitis and poliomyelitis-like paralysis [3]–[10].

Human enterovirus 71 was identified for the first time in the USA in 1969, and since then EV71-associated neurological disease has been observed in outbreaks throughout the world [1], [2], [4], [8], [11]–[17]. On the basis of VP1 nucleotide sequence comparisons, three genogroups of EV71 have been defined, designated A, B and C, showing a genetic divergence of about 17% at the nucleotide level [18]–[20]. BrCr strain, isolated in the USA in 1969 [19], is the reference strain of Genogroup A. Genogroups B and C are more commonly reported, and consist of subgenogroups B0 to B5 and C1 to C5 respectively [19]–[25].

The incidence of EV71 infection seems to have increased in the Asian Pacific region since 1997. Several countries within this region have been affected by one or more massive outbreaks of EV71 with ten to hundreds of thousands of cases of HFMD and hundreds of fatal cases as a consequence of neurological disease. Typing of EV71 strains revealed that these outbreaks were caused by strains belonging to distinct, recently emerged EV71 subgenogroups (B3–B5, C2, C4, C5) [3], [6], [8], [12], [15], [20], [23], [25]–[27].

In China, the first outbreak of HFMD caused by EV71 was reported in Shandong province in 2007 [28], followed by a widespread outbreak across the country in 2008. In contrast with trends observed in other Asian countries, the numbers of reported EV71 infections in China have been continuously high since that time [16], [27]. In Guangdong province alone, over 200,000 patients were identified, of which about one hundred died between 2008 and 2010 [27]. However, drawing conclusions on the basis of surveillance data alone is difficult, because surveillance systems depend on compliance to notification by physicians, which in turn depends on many factors. Therefore, we sought to explore the use of molecular virological data analysis to gain deeper insight in the epidemiological behavior of EV71 in China. We studied EV71 genetic diversity using the VP1 genes of 257 EV71 strains collected in Guangdong province, where the first strain of EV71 isolated in maninland China, from 2008 to 2010 as part of HFMD surveillance. We supplemented these with 305 GenBank EV71 C4 reference strains collected in China from 1998 to 2010. Viral population dynamics of EV71 in China from 1998–2010 were also analyzed to study whether the increase in number of reported EV71 infections reflects a real increase in viral spread or is just the result of increased awareness.

## Results

### VP1 Nucleotide Sequence Comparison

**Figure 1:**
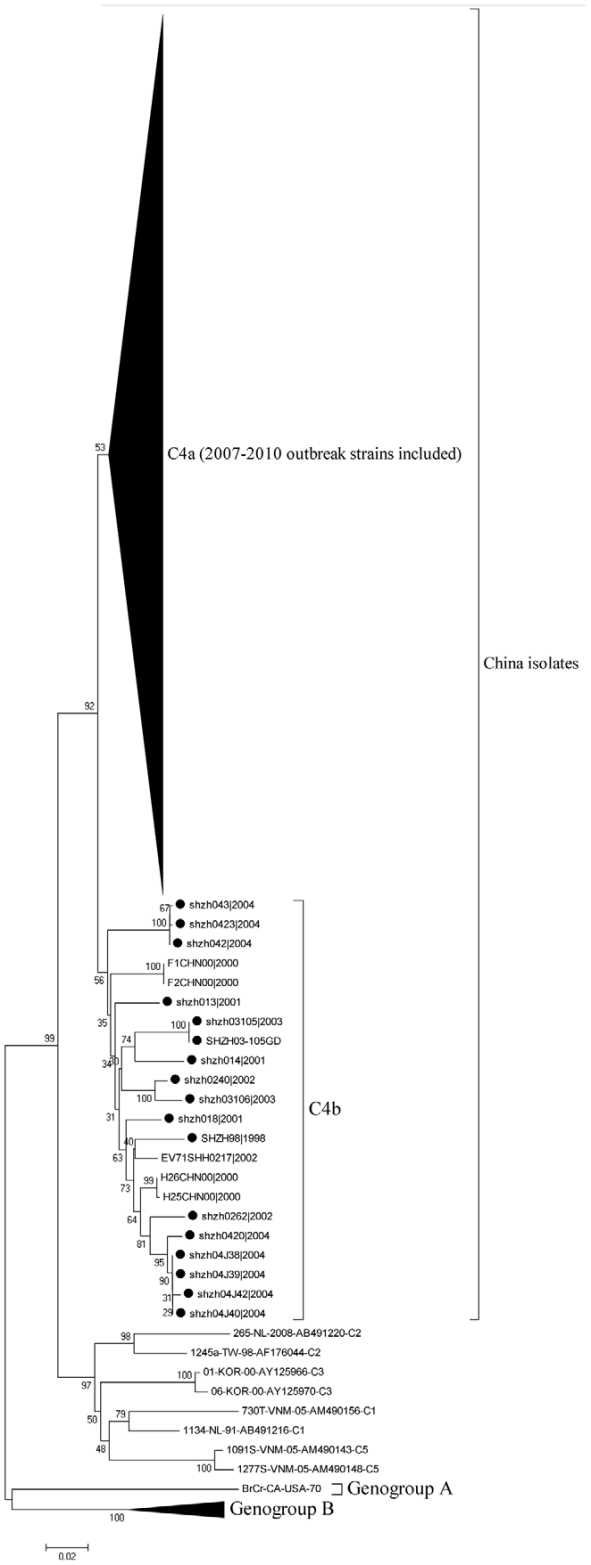
Phylogenetic relationship among EV71 strains isolated in mainland China and those downloaded from GenBank. The total 562 EV71 strains were isolated in mainland China from 1998 to 2010 and other genogroup A, genogroup B and subgenogroup C1, C2, C3, C5 strains were downloaded from GenBank. The phylogenetic tree was generated by using the neighbor-joining method based on alignment of complete VP1 gene sequences. The bootstrap values of 1000 replicates for major branches are displayed as numbers at the nodes. Strains isolated in Shenzhen from 1998 to 2004 were marked by the sombol •.

**Figure 2:**
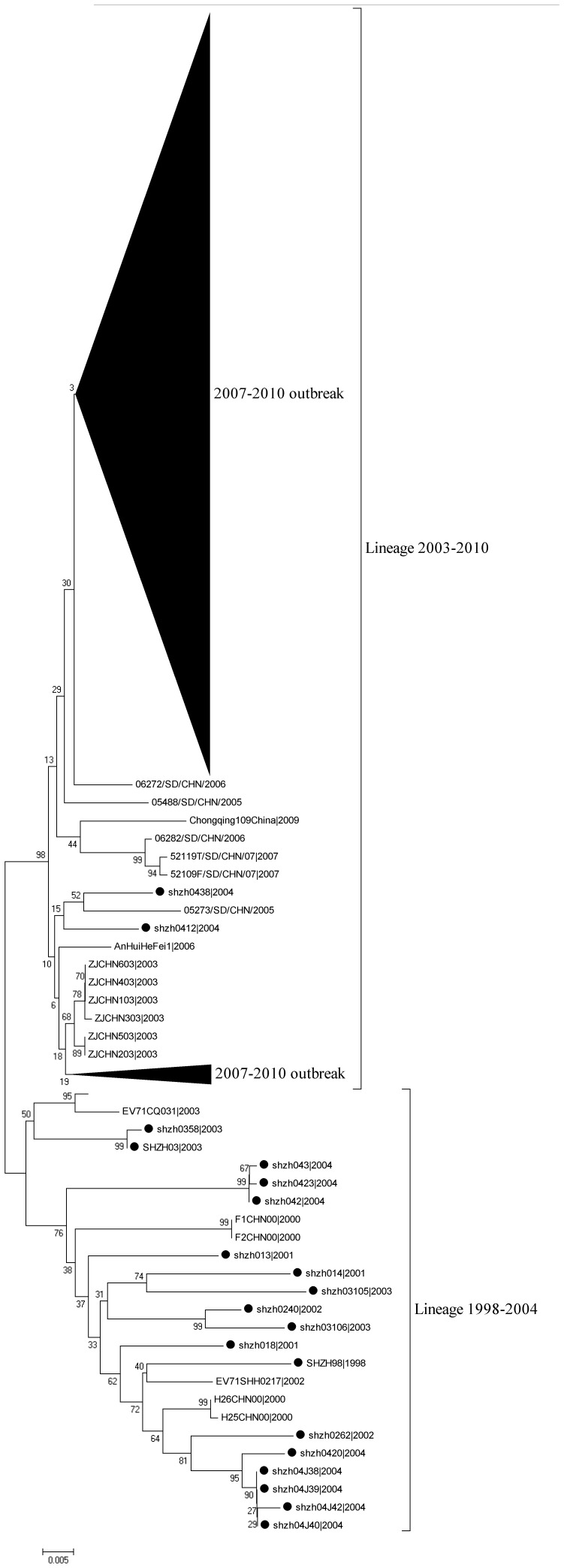
Phylogenetic relationship among the 562 EV71 C4 strains isolated in mainland China from 1998 to 2010. 257 China strains are from the present study, and 305 China strains are from GenBank. The bootstrap values of 1000 replicates for major lineages are displayed as numbers at the nodes. Strains isolated in Shenzhen from 1998 to 2004 were marked by the sombol •.

For the 257 EV71 isolates, collected in Guangdong Province (China) as part of HFMD surveillance activities between 2008 and 2010, the complete VP1 encoding regions (891 nt) were amplified successfully and used to study genetic diversity of EV71 in China. Phylogenetic analysis by means of a neighbor joining method, showed that all the 562 Chinese isolates (1998–2010), including those of Guangdong (2008–2010), clustered within EV71 subgenogroup C4. This subgenogroup has been further subdivided into subcluster 4a and 4b. Guangdong strains isolated during the first outbreak in China in 2007 (Shangdong Province) and during the subsequent nationwide outbreak in 2008 to 2010 clustered into C4a (defined in a previous study [28], [29]) (Figure 1, 2). Remarkably, no representatives of the C4b cluster, most of which were composed of C4 reference strains isolated in Shenzhen City, Guangdong Province, China from 1998 to 2004, were found among the Guangdong isolates from 2008 to 2010.

### Viral Population Dynamics

**Figure 3:**
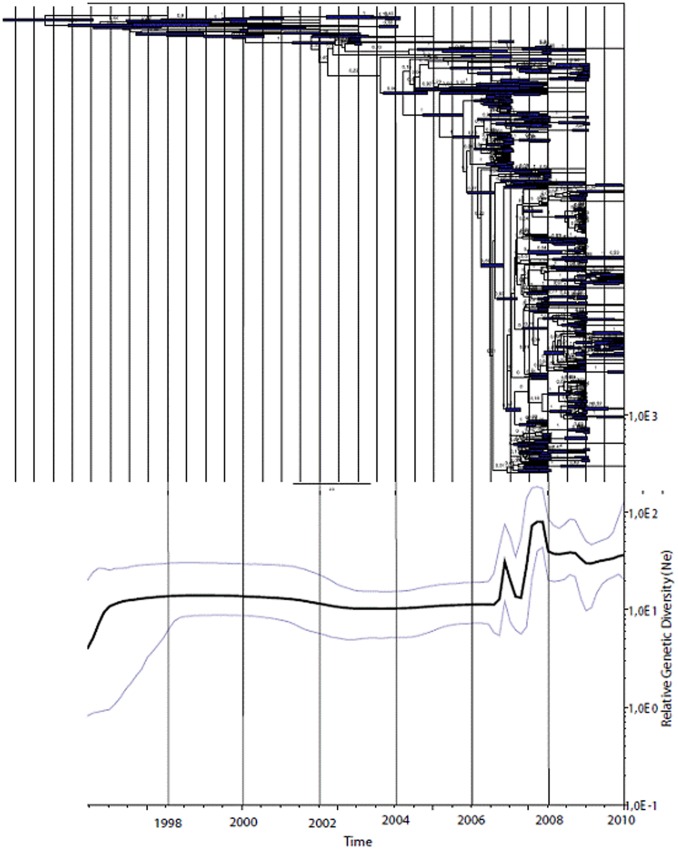
Maximum Clade Credibility tree and Bayesian Skyline of the China EV71 C4 strains. The upper portion of the figure was maximum clade credibility tree with 95% highest posterior density (HPD) intervals for the node times (in years) and posterior probabilities for branching events. Tree was generated by the MCMC method in BEAST on the basis of a multiple alignment of VP1 nucleotide sequences of EV71 C4 strains collected in Guangdong Province from 2008 to 2010 and Genbank EV71 C4 reference strains isolated in mainland China from 1998 to 2010. Below the tree, the Bayesian Skyline with 95% HPD intervals shows the relative measure for genetic diversity through time (values plotted on y-axis).

Viral population dynamics were estimated over time using Bayesian coalescent analysis of the VP1 nucleotide sequence alignment of the Guangdong isolates and GenBank C4 reference strains isolated in China from 1998 to 2010 (n = 562) [30]. A measure of coalescence rate or relative genetic diversity through time was estimated using a Bayesian skyline plot model [31]. The Bayesian skyline plot employs a piecewise-constant model to describe the change in effective population size through time. Up to 2006, a constant genetic diversity of EV71 was observed, suggesting stable endemic circulation of the virus in China in the first part of the study period (Figure 3). Just before 2007 and once again just before 2008, a sharp elevation in the genetic diversity was observed suggesting an exponential growth of the virus population. Genetic diversity decreased in 2009 and 2010 and showed a stable equilibrium, but remained higher than in the period before 2008. Regarding the Maximum Clade Credibility (MCC) tree, generated during the BEAST analysis, the phylogeny of C4 showed a ladder-like structure suggesting a continual replacement of lineages through time. The 2009 and 2010 strains appear to have further evolved from the 2008 outbreak strains.

### Amino Acid Sequence Comparisons

**Figure 4:**
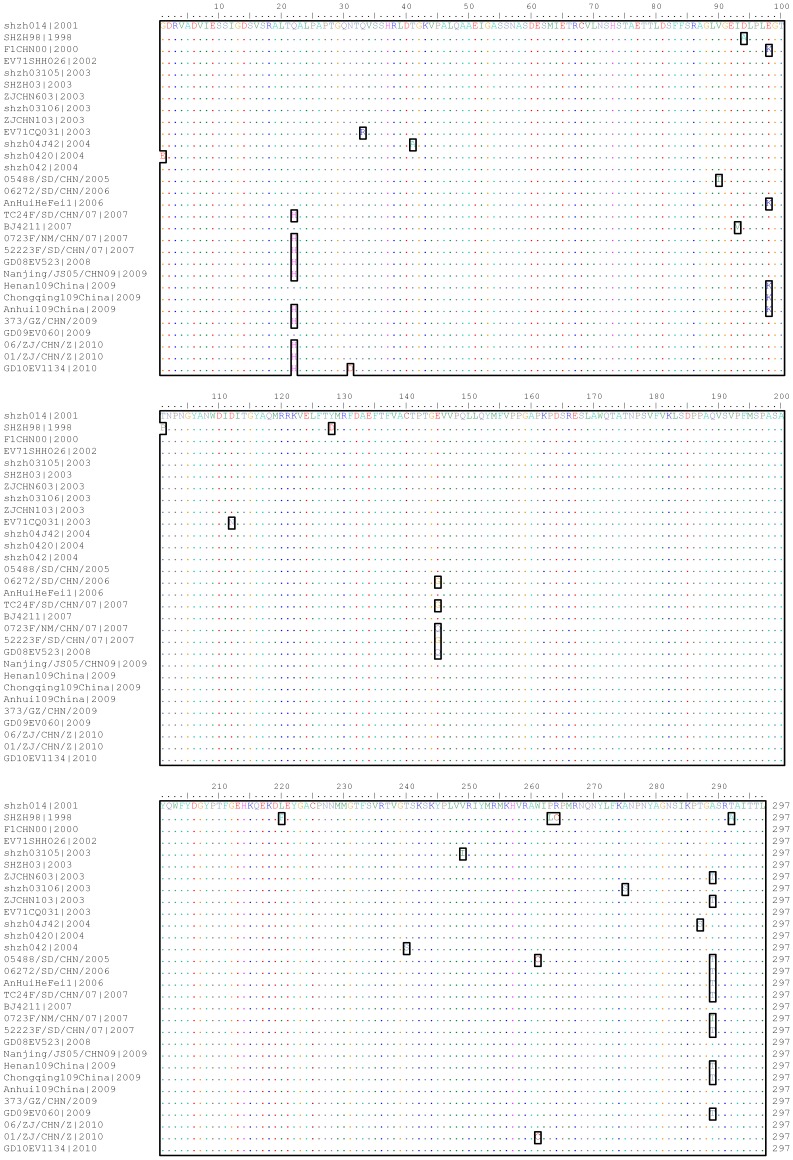
Alignment of deduced amino acid sequences of the VP1 fragment of representative EV71 strains collected in mainland China from 1998 to 2010. The amino acid substitutions are marked by using small panes.

To study a potential role of antigenic drift in the increased viral spread just before 2007, and once again just before 2008, VP1 encoding regions were used for amino acid sequence comparisons. Considering mutations that distinguish isolates from years with increased viral spread from those of other years, C4 viruses of 1998–2006 differed from 2007–2010 isolates in residue 22 (Q22H), of which the percentage of glutamic (Q) were 100% and 10.69% respectively. In addition, there were reverse mutations of amino acid in the VP1 gene. For instance, 2007 strains showed high variability in residue 145 (Q/G/E), with the percentage of 16.18%, 26.47% and 57.35% respectively, whereas C4 viruses of 1998–2006 and 2008–2010 had glutamic acid residue (E) at position 145 with almost the same percentage of 97.37% and 97.15% respectively (Figure 4). We also found reverse mutation in residue 289. The percentage of alanine (A) and threonine (T) were 72.73% and 27.27% respectively in the time period of 1998–2004, they changed to 22.22% and 77.78% respectively from 2005–2007, and later reverted to 79.21% and 20.79% respectively in the time period of 2008–2010 (*P*<0.001).

## Discussion

This study describes the genetic diversity of the VP1 genes of EV71 strains isolated in Guangdong Province. Samples were originally collected from 2008 to 2010 as part of HFMD surveillance activities in relation to genetic diversity observed in Mainland China since 1998. Up to present, all EV71 strains isolated in China belong to subgenogroup C4 and could be separated into two previously defined clusters, C4a and C4b [28], [29], which is rather different from that of other Asia Pacific countries [3], [6], [13]. Guangdong isolates phylogenetically interspersed with C4 reference strains isolated from other parts in Mainland China from 2003 to 2010, suggesting that the epidemiology of EV71 has a nationwide nature.

Phylodynamic analyses indicated that the increased reporting of EV71 infections in China since 2007 reflects a real increase in viral spread and is thus not a result of changes in surveillance setup or increased awareness. One could speculate that increases in virus population size coincide with an increased sampling density. The coalescence rates for the samples obtained in 2007 and 2008, however, were extremely rapid, with most lineages quickly coalescing to a single recent common ancestor, strongly indicating true exponential growth of the virus population. The analyses were performed using several different nucleotide substitution models and molecular clock models. All of these showed similar topology in MCC trees and similar trends in bayesian skyline plots, indicating that model selection did not cause bias to the results presented in this study.

Different from those in other Asian countries, the viral spread and associated numbers of infections have remained persistently high in China since 2008. Regarding the epidemiologic behavior of EV71 in other Asian countries where successive outbreaks were caused by different EV71 subgenogroups, the question arises why C4 is able to persist in mainland China. The persistence could be explained by a circulation network in which EV71 continuously hops from one epidemic to the other. Such a network has previously been shown for circulation of seasonal Influenza A (H3N2) viruses in East and Southeast Asia [32]. Another explanation could be found among study results published by Tee *et al.* [33], who demonstrated toggling of VP1 amino acid residues 145, 262 and 289 through time on the core trunk branches of C1 phylogenies. As observed for C1 viruses, the phylogeny of subgenogroup C4 viruses isolated in China showed a ladder-like structure suggesting a continual replacement of lineages through time (Figure 3). Our C4 sequence data set showed variation of residues 145 and 289 among others through time as well, which were similar to the study results published by Tee *et al.* [33], possibly explaining why C4 is able to persist among the Chinese population. There were some new mutations that differed from those previous study results, such as residue 22. Further study including antigenic characterization of these C4 variants using serum neutralization assays will be needed to test this hypothesis. This information will be of value for development of EV71 vaccines.

## Materials and Methods

### Ethics Statement

This work did not include direct contact with patients or volunteers, and research focused on previously collected samples, thus there was no need for ethical approval or informed consent. No identifying details were included in the article.

### HFMD Surveillance

In May 2008, HFDM was classified as a notifiable disease in China. From 2008 to 2010, a total of 288,274 cases (diagnosed on the basis of disease symptoms) were reported to the Center for Disease Control and Prevention of Guangdong (GDCDC) as part of HFMD surveillance. To clarify the etiologic agent, 2386 specimens, including stool, rectal swabs, vesicular swabs, cerebrospinal fluid, and throat swabs, were collected from 1944 patients including mild cases, severe cases and fatal cases from the 21 cities which cover the complete province, and subjected to a one-step enterovirus detection RT-PCR targeting the 5′untranslated region as described previously [34]. Enterovirus positive samples were cultured on Rhabdomyosarcoma cells and subsequently tested for presence of EV71 by an EV71 RT-PCR targeting the complete VP1 encoding region as described previously [28]. Of these, 257 EV71 strains isolated from 257 patients were included in the current study while samples of the other 1687 patients reacted EV71 negative.

### Characterization of EV71 RT-PCR Positive Samples

Purification of the RT-PCR products was performed by using the QIAquick® PCR Purification Kit (Qiagen, 2002). The nucleotide sequences of the EV71 genes were determined with the ABI Prism BigDye Terminator Cycle Sequencing Ready Reaction Kit version 3.1 (Applied Biosystems, Foster City, CA, USA) on an automated sequencer (Applied Biosystems model 3100), using the same EV71 VP1 sense and antisense primers [28].

### Phylogenetic Analysis

The VP1 gene of EV71 isolates were aligned together with the VP1 gene of the C4 reference strains of EV71 isolated in mainland China from 1998–2010 and other genogroup A, genogroup B and subgenogroup C1, C2, C3, C5 EV71 strains, available in GenBank (Table 1) using the Clustal W method implemented in MEGA software version 4.0 [35]. A Neighbor-joining (NJ) tree was constructed using the maximum composite likelihood model. One thousand bootstrap replicates were used to test the support for branches within the tree. Deduced amino acid sequences were compared by using BioEdit software version 7.09 [36].

**Table 1:** EV71 reference strains used in this study.

Strain	GenBank no.	Strain	GenBank no.
SHZH98|1998	AF302996	Fuyang5|2008	HQ694984
F1CHN00|2000	AB115490	Fuyang22|2008	EU913466
H26CHN00|2000	AB115493	BJ08Z0255|2008	FJ606450
H25CHN00|2000	AB115492	BJ08Z0201|2008	FJ606449
F2CHN00|2000	AB115491	BJ08Z0114|2008	FJ606448
shzh018|2001	AY895091	BJ08Z0043|2008	FJ606447
EV71SHH026|2002	AY547499	Chen1/GZ/CHN/2008	GU190181
EV71SHH0217|2002	AY547500	168/GZ/CHN/2008	GU190178
SHZH03|2003	AY465356	152/GZ/CHN/2008	GU190177
ZJCHN603|2003	AY905619	145/GZ/CHN/2008	GU190176
ZJCHN503|2003	AY905618	142/GZ/CHN/2008	GU190175
ZJCHN403|2003	AY905617	136/GZ/CHN/2008	GU190174
ZJCHN303|2003	AY905616	133/GZ/CHN/2008	GU190173
ZJCHN203|2003	AY905615	130/GZ/CHN/2008	GU190172
ZJCHN103|2003	AY905614	129/GZ/CHN/2008	GU190171
EV71CQ031|2003	AY547501	122/GZ/CHN/2008	GU190170
AnHuiHeFei1|2006	EU697903	1/GZ/CHN/2008	GU190169
BJ4211|2007	EU024958	DTID/ZJU74|2008	FJ158601
BJ4243|2007	EU019910	DTID/ZJU62|2008	FJ158600
0723F/NM/CHN/07|2007	EU910869	EV71/Zhejiang08|2008	EU864507
0718F/NM/CHN/07|2007	EU910868	AnHuiFuYang17|2008	EU697902
0717F/NM/CHN/07|2007	EU910867	AnHuiFuYang12|2008	EU697901
0716F/NM/CHN/07|2007	EU910866	EV71/Lanzhou10|2008	GQ855294
0715F/NM/CHN/07|2007	EU910865	EV71/Lanzhou09|2008	GQ855293
0712F/NM/CHN/07|2007	EU910864	EV71/Lanzhou08|2008	GQ855292
0711F/NM/CHN/07|2007	EU910863	EV71/Lanzhou07|2008	GQ855291
0709F/NM/CHN/07|2007	EU910862	EV71/Lanzhou06|2008	GQ855290
0708T/NM/CHN/07|2007	EU910861	EV71/Lanzhou05|2008	GQ855289
EV71/FuyangAnhuiPRC/1708/3|2008	EU703814	EV71/Lanzhou04|2008	GQ855288
EV71/FuyangAnhuiPRC/1708/2|2008	EU703813	EV71/Lanzhou03|2008	GQ855287
EV71/FuyangAnhuiPRC/1708/1|2008	EU703812	EV71/Lanzhou02|2008	GQ855286
CY44/BJ/CHN/2008	FJ469161	EV71/Lanzhou01|2008	GQ855285
CY43/BJ/CHN/2008	FJ469160	Henan109China|2009	GU196833
Cy29/BJ/CHN/2008	FJ469159	Henan209China|2009	GQ994992
CY28/BJ/CHN/2008	FJ469158	Chongqing309China|2009	GQ994991
CY21/BJ/CHN/2008	FJ469157	Chongqing209China|2009	GQ994990
CY20/BJ/CHN/2008	FJ469156	Chongqing109China|2009	GQ994989
CY17/BJ/CHN/2008	FJ469155	Anhui109China|2009	GQ994988
CY15/BJ/CHN/2008	FJ469154	373/GZ/CHN/2009	GU190179
CY11/BJ/CHN/2008	FJ469153	06/ZJ/CHN/Z|2010	HM855955
CY6/BJ/CHN/2008	FJ469152	05/ZJ/CHN/Z|2010	HM855954
HN08HLF3|2008	GQ121134	04/ZJ/CHN/Z|2010	HM855953
HN08HLF2|2008	GQ121133	03/ZJ/CHN/Z|2010	HM855952
Xinhui9|2008	EU999179	02/ZJ/CHN/Z|2010	HM855951
Xinhui8|2008	EU999178	01/ZJ/CHN/Z|2010	HM855950
Xinhui7|2008	EU999177	265-NL-2008	AB491220
Zhuhai152|2008	EU999176	1245a-TW-98	AF176044
Zhuhai164|2008	EU999175	01-KOR-00	AY125966
Zhuhai171|2008	EU999174	BrCr-CA-USA-70	U22521
Zhuhai213|2008	EU999173	9923-SYD-01	AY940107
ZhuhaiJC498|2008	EU999172	2027-SIN-01	AF376111
ZhuhaiJC455|2008	EU999171	06-KOR-00	AY125970
ZhuhaiJC467|2008	EU999170	730T-VNM-05	AM490156
Fuyang49|2008	EU913471	1134-NL-91	AB491216
Fuyang31|2008	EU913470	1091S-VNM-05	AM490143
Fuyang44|2008	EU913469	1277S-VNM-05	AM490148
Fuyang26|2008	EU913468	203 reference strains*	

* The GenBank numbers of the 203 reference strains are AY895129-AY895145, EU753363 - EU753418, FJ765416 - FJ765435, GQ121417 - GQ121441, GQ253391 - GQ253423, GQ487666 - GQ487689 and GU353079 - GU353106. All of these strains are part of the 305 GenBank EV71 C4 reference strains collected in Mainland China from 1998 to 2010.

### Nucleotide Sequence Accession Numbers

The nucleotide sequence data reported in this paper will appear in the DDBJ/EMBL/GenBank nucleotide sequence databases with the accession numbers JN579717-JN579942, JF519696-JF519719.

### Viral Population Dynamics

On the basis of VP1 nucleotide sequences of EV71 strains isolated in Guangdong Province from 2008 to 2010 (n = 257), and EV71 C4 reference strains isolated in China from 1998 to 2010 and downloaded from GenBank (n = 305, Table 1), viral population dynamics over time were estimated using a Bayesian Markov Chain Monte Carlo approach (MCMC, implemented in BEAST software version 1.5.4) that incorporates the date of virus sampling [30]. Preliminary analyses using multiple combinations of nucleotide substitution models and molecular clock models revealed that the Hasegawa-Kishino-Yano (HKY) nucleotide substitution model with a discrete gamma distribution (accommodating rate variation among sites in the alignment) in combination with the strict molecular clock (assuming that mutation rates are similar among all branches) resulted in the most optimal convergence of posterior probabilities [37]. Furthermore, we partitioned alignment sites into first and second codon positions, and third codon positions, respectively, to allow different rates of substitution for the 1^st^ +2^nd^ versus the 3^rd^ codon position. To infer the dynamics of EV71 genetic diversity through time, we employed a Bayesian skyline plot model [31]. We specified 20 groups of coalescent intervals to capture the past population dynamics in the piecewise constant demographic function. The posterior distribution for the Bayesian skyline plot parameters yields the most plausible piecewise constant expectations for the coalescence rates through time in the genealogies, which in turn, represent the most plausible evolutionary histories for the sequence data. The MCMC analysis was run for 100,000,000 generations. Stationarity and mixing efficiency were examined using Tracer (http://tree.bio.ed.ac.uk/software/tracer/).
